# Nasal sprayed particle deposition in a human nasal cavity under different inhalation conditions

**DOI:** 10.1371/journal.pone.0221330

**Published:** 2019-09-06

**Authors:** Hadrien Calmet, Kiao Inthavong, Beatriz Eguzkitza, Oriol Lehmkuhl, Guillaume Houzeaux, Mariano Vázquez

**Affiliations:** 1 Barcelona Supercomputing Center (BSC-CNS), Department of Computer Applications in Science and Engineering, Barcelona, Spain; 2 School of Engineering (Mechanical & Automotive), RMIT University, Bundoora, Victoria, Australia; Heidelberg University, GERMANY

## Abstract

Deposition of polydisperse particles representing nasal spray application in a human nasal cavity was performed under transient breathing profiles of sniffing, constant flow, and breath hold. The LES turbulence model was used to describe the fluid phase. Particles were introduced into the flow field with initial spray conditions, including spray cone angle, insertion angle, and initial velocity. Since nasal spray atomizer design determines the particle conditions, fifteen particle size distributions were used, each defined by a log-normal distribution with a different volume mean diameter (*Dv*50). Particle deposition in the anterior region was approximately 80% when *Dv*50 > 50μm, and this decreased to 45% as *Dv*50 decreased to 10μ m for constant and sniff breathing conditions. The decrease in anterior deposition was countered with increased deposition in the middle and posterior regions. The significance of increased deposition in the middle region for drug delivery shows there is potential for nasal delivered drugs to reach the highly vascularised mucosal walls in the main nasal passages. For multiple targeted deposition sites, an optimisation equation was introduced where deposition results of any two targeted sites could be combined and a weighting between 0 to 1 was applied to each targeted site, representing the relative importance of each deposition site.

## Introduction

The nasal cavity is a promising route for systemic drug delivery due to potential drug absorption through the porous endothelial membrane of the rich vascular capillary bed underneath the nasal mucosa [1, 2]. However, studies have shown many commercially available nasal spray devices deposit most of the atomized drug in the anterior portion of the nose [3, 4], and missing the nasal mucosa in the turbinate regions. Understanding deposition of atomized droplets within the main nasal passage provides insight effective nasal spray device design for targeted drug delivery.

Cheng et al. [5] studied particle deposition from nasal spray pumps on a multi-sectional nasal airway replica model and found that aerosols mainly deposited in the anterior regions, with a lesser amount in the turbinate regions on the inferior meatus. Smaller droplets, and narrowed spray angles allowed more droplets to deposit in the main nasal passage. Suman et al. [6] investigated deposition patterns in relation to in vitro measurements of two nasal spray pumps with different performance characteristics and found that spray angle and plume geometry did not affect droplet deposition in the nose. It was stated that large spray cone angles produced a spray pattern greater than the width of the nasal passage and was unlikely to alter the distribution of droplets in the nose because the spray would impinge on the vestibule walls, with only the droplets that are aligned with the nasal valve opening able to enter the main nasal passage. Furthermore, a full spray plume would never can freely develop in the nasal cavity as it would in an unconfined space.

Guo et al. [7] showed that a low viscosity nasal spray formulation with a wider plume angle (69°) and small volume median diameter (*Dv*50 = 47 − 86μm) enhanced deposition in the main nasal passage compared to a higher viscosity formulation (32° plume angle; *Dv*50 = 100 − 130μm). The liquid viscosity was the most significant influence on droplet size due to the liquid breakup during atomization. Foo et al. [8] found that both spray cone angle and administration angle were important factors in determining deposition efficiency, where sprays with small cone angles were capable of reaching 90% deposition efficiency in the main nasal passage. It also found that particle size, viscosity, and inspiratory flow rate had relatively minor influence on deposition within the nasal cavity.

Other experimental studies include [9] that found greater anterior deposition in children (12 year old model) leading to decreased effectiveness; Pu et al. [10] investigated the effect of spray formulation (e.g. viscosity) on deposition patterns in a nasal cavity cast; Warken et al. [11] found the optimum administration angle for nasal sprays applied to ten 3D-printed nasal cavity replicas, and showed that it could increase deposition in the main nasal passage. They also suggested inhalation flow had no significant effect on the deposition pattern, based on steady flows of 10 and 60 L/min. However this may differ if unsteady inhaled flow or a sniff was used. Characterisation of the nasal spray was performed experimentally with measurements of external characteristics of unsteady spray atomization from a nasal spray device [12], and measurements of droplet size distribution and analysis of nasal spray atomization from different actuation pressure [13]. Newman et al. [14] evaluated the role of in-vitro and in-vivo methods of two similar nasal pump sprays, where significant differences in in-vitro parameters were not reflected in differences in nasal deposition in-vivo. This suggests that any in-vivo studies (and to some extent in-silico studies) should expect differences in results.

Computational studies of sprayed particle deposition in the human nasal cavity have found relationships for deposition efficiencies or penetration into the turbinate mucosa of the main nasal passage, with nasal spray parameters, such as spray cone angle and the particle size distribution produced. Kimbell et al. [15] found that particle penetration past the nasal valve improved when particle inertia was reduced through smaller particle diameters or reduced spray velocity. The positioning of 1cm into the nostril, with inspiratory flow present was recommended. Inthavong et al. [3, 16] found that if the particles exhibited increased tangential velocities (through swirling) as it exited the spray nozzle, then increased deposition in the turbinate region could be achieved. This assumed a hollow cone spray which is the typical spray formation produced from pressure-swirl atomizers.

Other computational studies include: Keeler et al. [17] who investigated the influence of nasal airway geometry from different ethnic groups on spray particle deposition and found that white and Latin Americans had the least patent nasal cavity, although this was based on four models per ethnic group; Kiaee et al. [18] that found particle diameter and particle injection speed, were the dominant parameters (other parameters were spray cone angle, spray release direction, and particle injection location) that influenced deposition in seven adult nasal airways; and Fung et al. [19] performed CFD modelling to characterise the nasal spray atomization stage. Djupesland et al. [20] showed a bi-directional nasal delivery concept reduced lung deposition by taking advantage of the posterior connection between the nasal passages persisting when the soft palate automatically closes during oral exhalation.

The influence of breathing conditions during spray delivery on the uptake of the drugs are unknown. Inhalation can be a short burst of high flow rate (as a sniff), a shallow steady breath, or zero breathing found in a breath hold. However, many computational studies with drug delivery have only applied a steady flow condition. For example studies have used laminar steady inspiratory flow rates [3, 4, 17, 18] when the flow rate was approximately <15L/minor, and steady RANS (Reynolds Averaged Navier-Stokes) turbulent flows when the flow rate was >20L/min [16, 21]. While there are many ways to inhale during drug delivery, this study looked at the influence of a sniff compared to commonly used steady flow rate, and a zero inhalation flow on particle deposition in the human nasal cavity.

To increase the accuracy of the work, high fidelity LES turbulence model was used to account for the transient, and unstable nature of the fluid-particle dynamics in the flow field. While the RANS models provide efficient computations, studies showed the fluid-particle turbulent dispersion coupling required empirically based adjustments [22–24]. To avoid this, the Large Eddy Simulation (LES) method was used for the transient, and turbulent flow behaviour as provides anisotropic turbulent fluctuations which are transferred directly onto the particles.

Studies employing LES techniques include: Bates et al. [25] assessed the relationship between movement and airflow in the upper airway motion determined from magnetic resonance imaging; Farnoud et al. [26] and Payri Marin et al. [27] used the LES model to deliver mono-disperse particles with the diameters of 2.4 and 10 μm uniformly and randomly injected at the nostrils with constant inhalation flow rates of 4.78 L/min and 7.5 L/min; Covello et al. [28] studied droplet deposition for different sizes of water droplets on a patient-specific anatomy under steady inspiration at two breathing intensities; Bahmanzadeh et al. [29] investigated airflow and micro-particle deposition in human nasal airway pre- and post-virtual sphenoidotomy surgery; Mylavarapu et al. [30] inspected the airflow through the nasal cavity comparing its results to experimental pressure drop measurements; and Li et al. [31] evaluated the performance of various turbulence models including the LES model for airflow through a nasal cavity. It’s expected that the results using LES method will add to the existing literature regarding nasal spray device performance.

## Methods

### Ethics statement

The protocol of the study has been approved by the ethical committee of King’s College Hospital of London. The subject signed informed consent before recruited in the study.

### Nasal geometry and mesh generation

**Fig 1:**
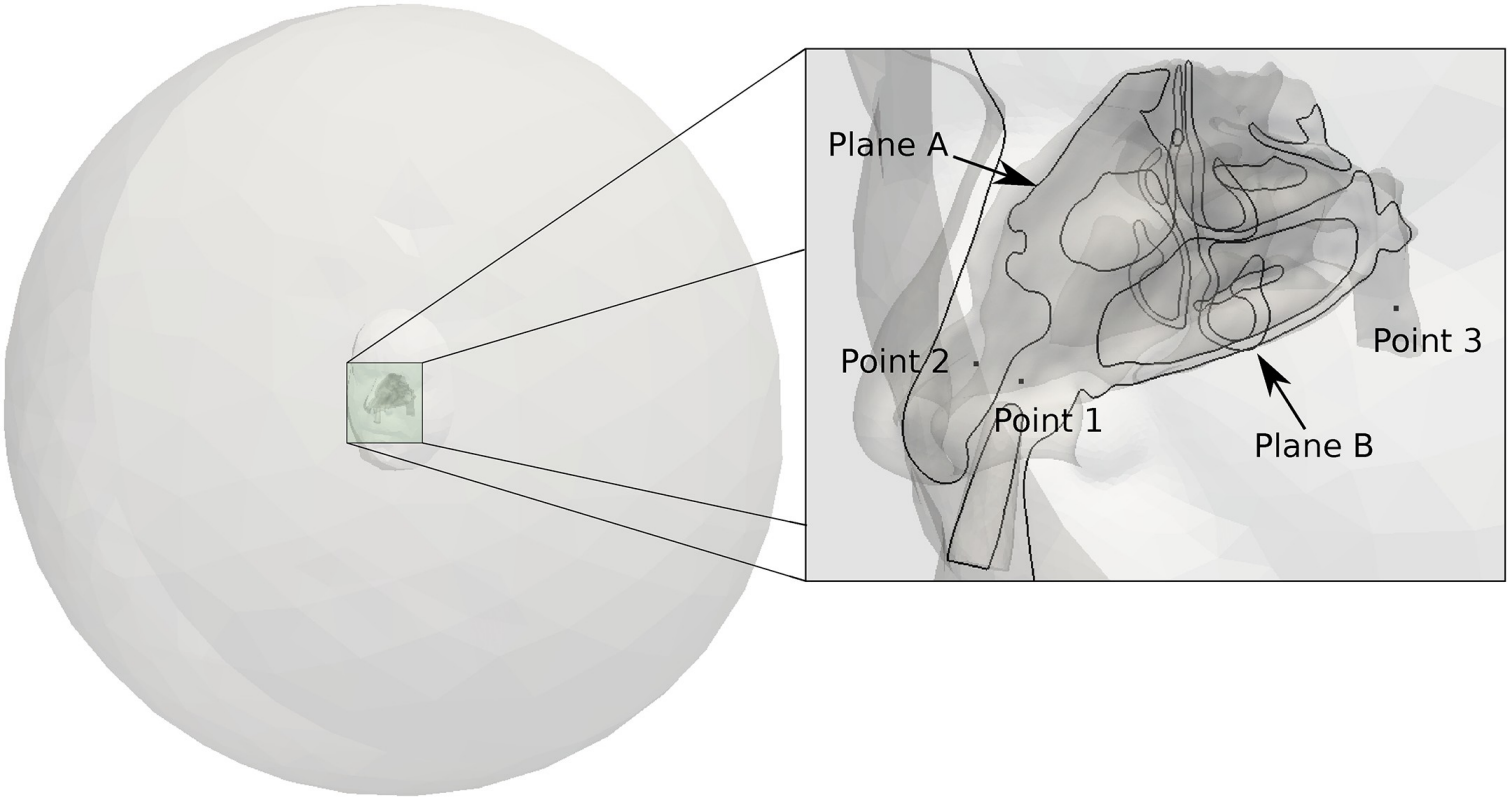
Geometry description details of planes, and points used in this study. Plane A: sagittal section through left nasal cavity; Plane B: coronal section through nasal cavity; Point 1 is located in the left vestibule; Point 2 on the right vestibule; and Point 3 in the posterior nasopharynx.

The geometry was constructed from retrospective computed tomography scans of a healthy 48-year-old male patient, collected from a large hospital database. The scan was performed in the supine position and the resulting data set comprises of 912 images in the axial plane, with 1 mm slice thickness and in-plane 0.65 × 0.65 mm pixel size. A consultant radiologist reported the nasal airways as clear and of normal appearance. Further details of the patient and the segmentation are given in [32]. The patient’s face was surrounded by a hemisphere with radius of 0.5m (Fig 1) to insure a correct inflow condition at the nostril [33]. The geometry included a spray nozzle inserted into the left nostril which created an occluded nostril opening. The precise location of the nozzle is discussed later.

An unstructured mesh with prism layers was created using ANSYS-ICEM-CFD (Ansys Inc., USA). The octree-based method generated a fine resolution surface mesh with surface smoothing to maintain smooth transitions between mesh sizes. The volume was filled with tetrahedral cells with a smooth cell transition ratio of 1.2 employed close to the boundary wall, using the Delaunay method. The final step inserted prism layers at the near wall boundaries to resolve high velocity gradients at the wall. Three different mesh sizes were created with the details given in Table 1. The coarsest mesh (M1), had 2.3 million elements, the medium mesh (M2) had 6.3 million elements and the fine mesh (M3) had 50.2 million elements.

**Table 1:** Summary of different mesh resolutions and simulation parameters with *N*_*N*_: Number of nodes, *N*_*E*_: Number of elements, Δ*t*: Time step, Δ: Grid size, *N*_*pl*_: Number of prism layers and *h*_*pl*_: Height of total prism layer.

Mesh	*N*_*N*_(×10^6^)	*N*_*E*_(×10^6^)	Δ*t*(*μs*)	Δ(*mm*)	*N*_*pl*_	*h*_*pl*_(*mm*)
M1	0.6	2.3	5.8	0.8	3	0.6
M2	1.8	6.3	3.7	0.4	5	0.3
M3	14.2	50.1	1.2	0.2	10	0.3

**Fig 2:**
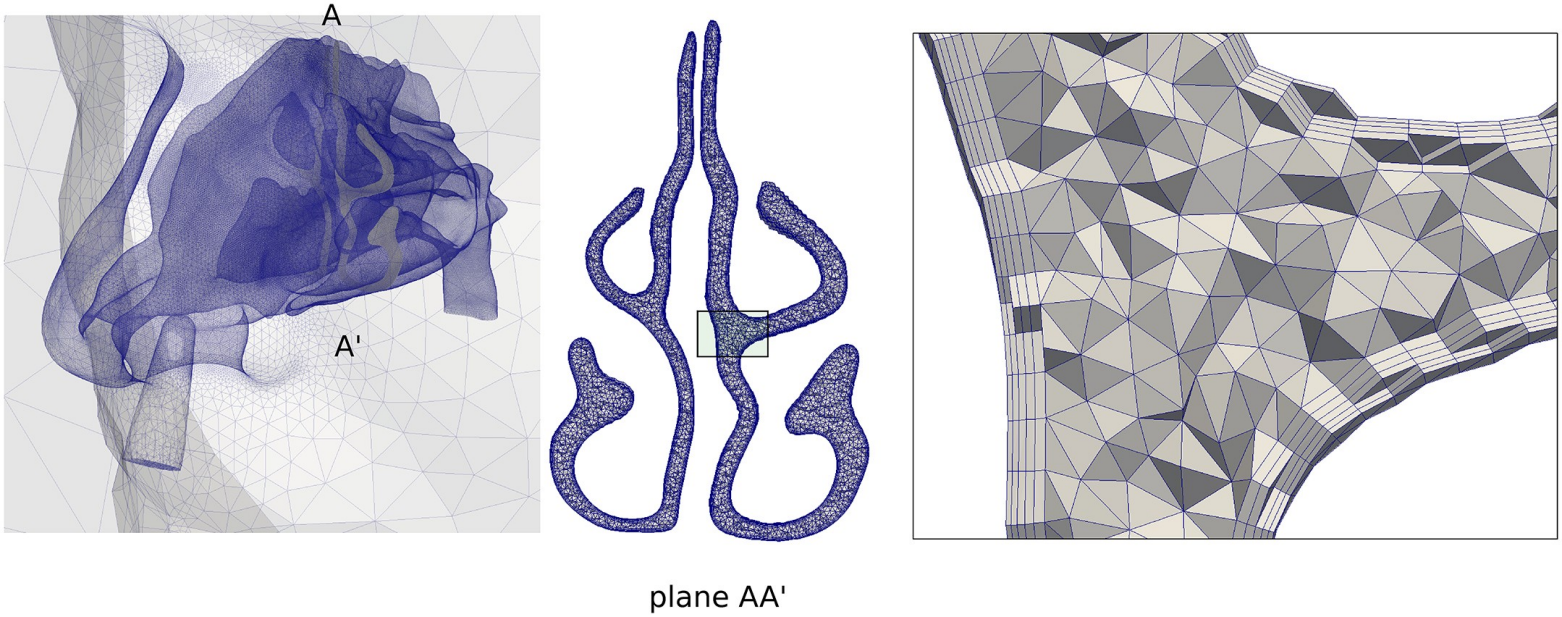
Grid generation topology for M2. Plane AA’ is the same as plane B, see Fig 1.

The topology of mesh M2 is presented in Fig 2. A minimum element height of 26μm at the wall was applied on the nasal valve, which is the most critical region. This produced a range of $y^{+}=\frac{u_{*}y}{\nu }=1\text{to}\;3$, for the most extreme case, which was the maximum velocity during sniffing, *Q*_*max*_ = 57.4L/min. The term u_*_ is the friction velocity at the wall, *y* is the distance to the wall and *ν* is the local kinematic viscosity of the fluid. The values of *y*^+^ are considered sufficiently small for resolving the near-wall flow dynamics [34] and falls into the range defined by [35] that describes well resolved wall-layers.

### Governing equation

#### Fluid solver

The fluid solver used the high performance computational mechanics code Alya [36], developed at Barcelona Supercomputing Center. The spatially filtered Navier-Stokes equations for a fluid moving in the domain Ω bounded by Γ = ∂Ω during the time interval (*t*_0_, *t*_*f*_) consist in finding a filtered velocity $\bar{u}$ and a kinematic pressure *p* such that

$$\begin{array}{c} \partial_{t}\bar{u}+\left(\bar{u}\cdot \nabla \right)\bar{u}-2\nu \nabla \cdot S\left(\bar{u}\right)+\nabla p-f=-\nabla \cdot \tau_{\text{ij}}\left(\bar{u}\right)\;\text{in}\;\Omega \times \left(t_{0},t_{f}\right), \end{array}$$ (1)

$$\begin{array}{c} \nabla \cdot \bar{u}=0\;\text{in}\;\Omega \times (t_{0},t_{f}), \end{array}$$ (2)

where *ν* is the fluid viscosity, **f** the vector of external body forces and $\text{S}(\bar{\text{u}})$ is the large-scale rate-of-strain tensor. In Eq 1 $\tau_{ij}(\bar{u})$ is the subgrid scale (SGS) stress tensor, which must be modelled. Its deviatoric part is given by

$$\begin{array}{c} \begin{array}{c} \tau_{\text{ij}}\left(\bar{u}\right)-\frac{1}{3}\tau_{\text{kk}}\left(\bar{u}\right)\delta_{\text{ij}}=-2\nu_{sgs}\nabla \cdot S\left(\bar{u}\right) \end{array} \end{array}$$ (3)

where *δ*_**ij**_ is the Kronecker delta. Suitable expression of the subgrid-scale viscosity, *ν*_*sgs*_ was used to close the formulation. The wall-adapting local-eddy viscosity model (WALE) [37] was applied. This model has demonstrated good results in simulations of respiratory airways which is comparable to more computational demanding models like the dynamic Smagorinsky model (see [38]).

To obtain the weak or variational formulation of the Navier-Stokes Eqs (1) and (2) the spaces of vector functions ${\text{V}}_{D}={\text{H}}_{D}^{1}\left(\Omega \right)$, ${\text{V}}_{0}={\text{H}}_{0}^{1}\left(\Omega \right)$ and *Q* = *L*^2^ (Ω) /ℜ. are introduced. *L*^2^ (Ω) is the space of square-integrable functions, *H*^1^ (Ω) is a subspace of *L*^2^ (Ω) formed by functions whose derivatives also belong to *L*^2^ (Ω), $H_{D}^{1}\left(\Omega \right)$ is a subspace of *H*^1^ (Ω) that satisfies the Dirichlet boundary conditions on Γ, $H_{0}^{1}\left(\Omega \right)$ is a subspace of *H*^1^ (Ω) whose functions are zero on Γ, and ${\text{H}}_{D}^{1}\left(\Omega \right)$ and ${\text{H}}_{0}^{1}\left(\Omega \right)$ are their vector counterparts in a 2 or 3 dimensional space.

For the evolutionary case **V**_*t*_ ≡ *L*^2^ (*t*_0_, *t*_*f*_; **V**_*D*_) and $Q_{t}\equiv D^{\prime }\left(t_{0},t_{f};Q\right)$ are introduced, where *L*^*p*^ (*t*_0_, *t*_*f*_; *X*) is the space of time dependent functions in a normed space *X* such that $\int_{t_{0}}^{t_{f}}{\left\|f\right\|}_{X}^{p}\;\text{d}t<\infty$, 1 < *p* < ∞ and *Q*_*t*_ consists of mappings whose *Q*-norm is a distribution in time. The weak form of problem (Eqs 1 and 2) with the boundary conditions is then: **u** ∈ **V**_*t*_, *p* ∈ *Q*_*t*_ such that

$$\begin{array}{c} (\partial_{t}\bar{u},v)+(\bar{u}\cdot \nabla \bar{u},v)+2\nu \;(S(\bar{u}),\nabla v)-(p,\nabla \cdot v)+(q,\nabla \cdot \bar{u})-(f,v)=(\tau_{\text{ij}}\left(\bar{u}\right),\nabla v), \end{array}$$

for all (**v**, *q*) ∈ **V**_0_ × *Q*. In the previous equations the convective form of the nonlinear term

$$\begin{array}{c} NL_{conv}(\bar{u})=\bar{u}\cdot \nabla \bar{u} \end{array}$$

was used, which is probably the most frequent choice in computational practice. Using Eq 2 other forms of the nonlinear term can be derived which are equivalent at the continuous level but have different properties at the discrete level. In the following we consider the skew-symmetric form

$$\begin{array}{c} NL_{skew}(u)=u\cdot \nabla u+\frac{1}{2}(\nabla \cdot u)u \end{array}$$

which has the advantage that it conserves kinetic energy at the discrete level and is commonly used in numerical analysis and DNS and LES simulations where energy conservation provides enhanced results (see for instance [39–41]).

A non-incremental fractional step method was used to stabilise pressure. This allowed the use of finite element pairs that do not satisfy the inf-sup conditions, such as equal order interpolation for the velocity and pressure used in this work. The set of equations is time integrated using an energy conserving Runge-Kutta explicit method lately proposed by [42] combined with an eigenvalue based time step estimator [43].

#### Particle transport and deposition modelling

Particle transport was simulated in a Lagrangian frame of reference, following each individual particle. The main assumptions of the model were:

- Particles were sufficiently small and the suspension was dilute to neglect their effect on airflow: i.e. one way coupling;
- Particles were spherical and do not interact with each other;
- Particle rotation was negligible;
- Thermophoretic forces were negligible;
- The forces considered were drag ***F***_*d*_, gravitational and buoyancy ***F***_*g*_;

Particles transport was predicted by solving Newton’s second law, and by applying a series of forces

$$\begin{array}{c} a_{p}=\left(F_{d}+F_{g}\right)/m_{p}. \end{array}$$ (4)

where ***x***_*p*_, ***u***_*p*_, ***a***_*p*_ are the particle position, velocity and acceleration, respectively; *m*_*p*_ is particle mass, *ρ*_*p*_ is density, *d*_*p*_ is diameter, and *V*_*p*_ is volume.

The equation for the drag force assumed the particle reached its terminal velocity and is given by

$$\begin{array}{c} F_{d}=-\frac{\pi }{8}\mu d_{p}C_{d}\text{Re}\left(u_{p}-u_{f}\right), \end{array}$$ (5)

where Re is the particle Reynolds number involving its relative velocity with the fluid:

$$\begin{array}{c} \text{Re}=\frac{|u_{p}-u_{f}|d_{p}}{\nu }. \end{array}$$

The drag coefficient used Ganser’s formula [44]:

$$\begin{array}{ccc} C_{d} & = & \frac{24}{\text{Re}\;k_{1}}\left(1+0.1118{\left(\text{Re}\;k_{1}k_{2}\right)}^{0.6567}\right)+0.4305\frac{k_{2}}{1+3305/(\text{Re}\;k_{1}k_{2})},\\ k_{1} & = & \frac{3}{1+2\psi^{-0.5}},\\ k_{2} & = & 10^{1.84148{\left(-{\text{log}}_{10}\left(\psi \right)\right)}^{0.5743}},\\ \psi & = & \text{sphericity,}\;(=1\;\text{for}\;\text{a}\;\text{sphere}). \end{array}$$

The gravity and buoyancy forces contribute to the dynamics of the particle when there is a density difference:

$$\begin{array}{c} F_{g}=V_{p}g\left(\rho_{p}-\rho \right), \end{array}$$

with ***g*** being the gravity vector.

Further details about particle transport and deposition modeling are available in [45, 46].

#### Particle deposition

The particle deposition efficiency was defined as:

$$\begin{array}{c} \eta_{depo}=\frac{N_{depo}}{N_{in}} \end{array}$$ (6)

where *N*_*dep*_ is the number of particles depositing on the surface area of interest, which can be the total nasal passage wall or local surface areas (e.g. olfactory regions), and *N*_*in*_ is the total number of particles released.

#### Boundary and initial conditions

The computational domain included a large hemisphere surrounding the patient’s face that ensured an accurate velocity field in the vicinity of the nostrils [33, 47]. A no-slip boundary condition was imposed on all airway walls and the flat surface of the hemisphere to mimic the effects of the face and body.

*Inflow condition*: Nasal spray medicines typically have more variable bioavailability than medicines delivered by other routes of administration because of variability associated with use (e.g. in the patient’s inspiratory flow pattern). While patients are instructed to breathe in slowly during the application, other modes of breathing can occur, such as a sniff, or a breath hold. Three different inhalation conditions were investigated. i) a sniff, labelled as profile A1; ii) constant flow rate, labelled as profile A2; and iii) breath hold, labelled as profile A3.

**Fig 3:**
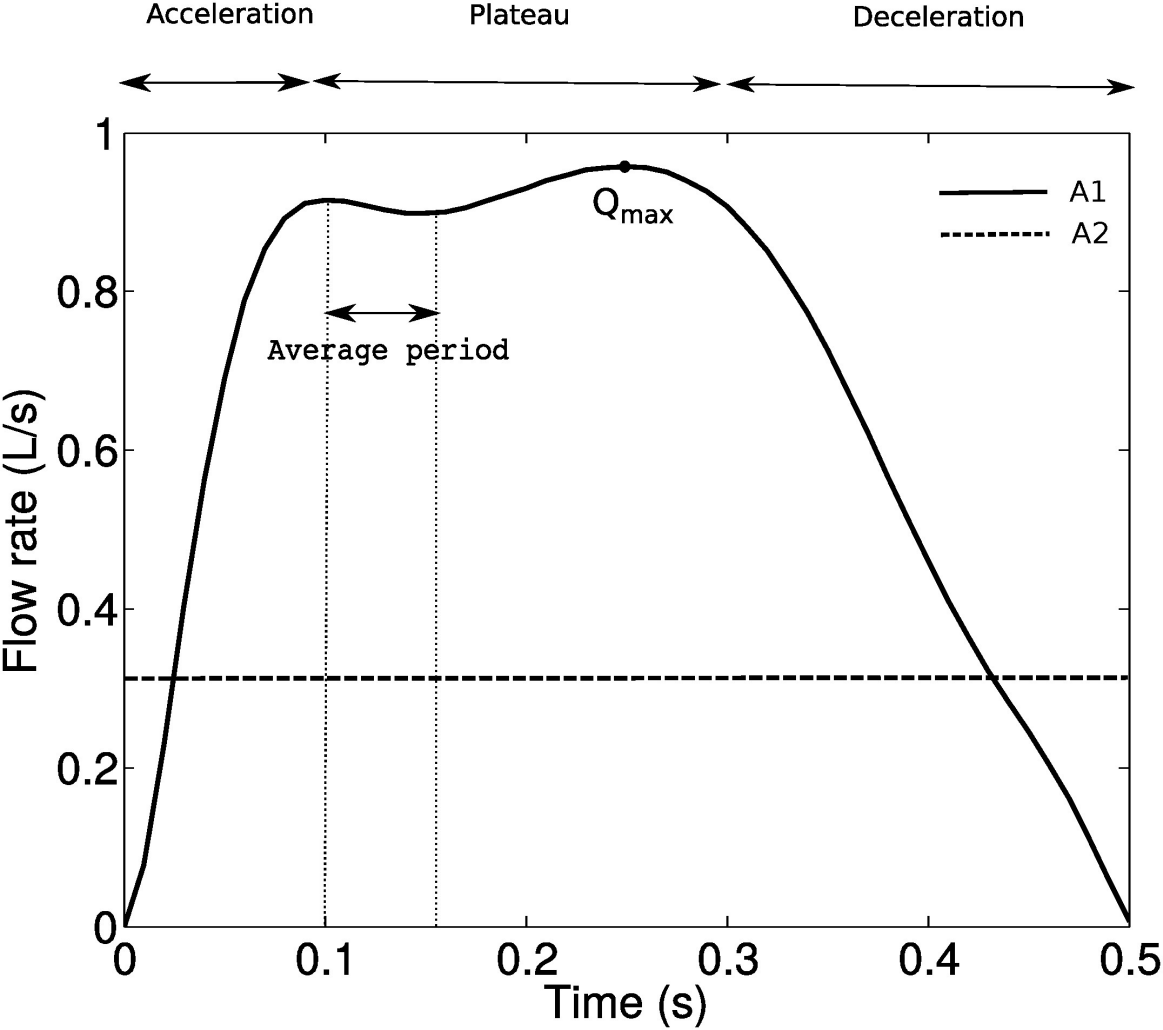
Flow rate profiles of different inhalation conditions, A1 and A2.

The sniff (profile A1) was in the form of a rapid and short inhalation, which was transient and prescribed as a time varying uniform velocity with direction normal to the hemisphere. To model the sniff, we used a polynomial function of order 10 derived from the experimental work detailed in [48], see S1 Appendix. The constant flow rate (profile A2) was set to *Q* = 20L/min. This is frequently used in the literature and considered as normal constant breathing inspiration. [45, 49, 50]. The non-breathing flow (profile A3) was created with a zero velocity field on the entire domain, which depicts a long holding breath with the assumption of no inhalation/exhalation effects. The inlet velocity profiles for A1 and A2 (Fig 3) were imposed as a Dirichlet condition on the hemisphere dome.

*Outflow condition*: A zero-traction outflow condition was imposed as a Neumann condition (the surface is free from external stress) at the outlet of the naso-pharynx.

#### Nasal spray

Experimental measurements of particle size distributions produced from nasal spray devices [51–54] were used as a base to define the initial particle conditions. Additional data from [15] was also used which included reported values of spray angles from 32° to 79°, averaged spray velocity 1.5 to 14.7 m/sec, and mean droplet size of 50*μ*m. The sprayed particle conditions in this study were: spray half cone angle = 40°; mean spray exit velocity = 18.0m/s; and a solid-cone type injection was assumed. The particle sizes were defined with a log-normal distribution defined through the probability density function

$$\begin{array}{c} f\left(x\right)=\frac{1}{\sqrt{2\pi }x\;\text{ln}\;\sigma_{g}}\text{exp}\;[-\frac{{\left(\text{ln}\;x-\text{ln}\;x_{50}\right)}^{2}}{2{\left(\text{ln}\;\sigma_{g}\right)}^{2}}] \end{array}$$ (7)

where *x* is the particle diameter. The log normal distribution median was initially set to *x*_50_, also know as *Dv*50 which is the volume median diameter, and ln *σ*_*g*_ is the standard deviation, where *σ*_*g*_ = 2.08.

**Fig 4:**
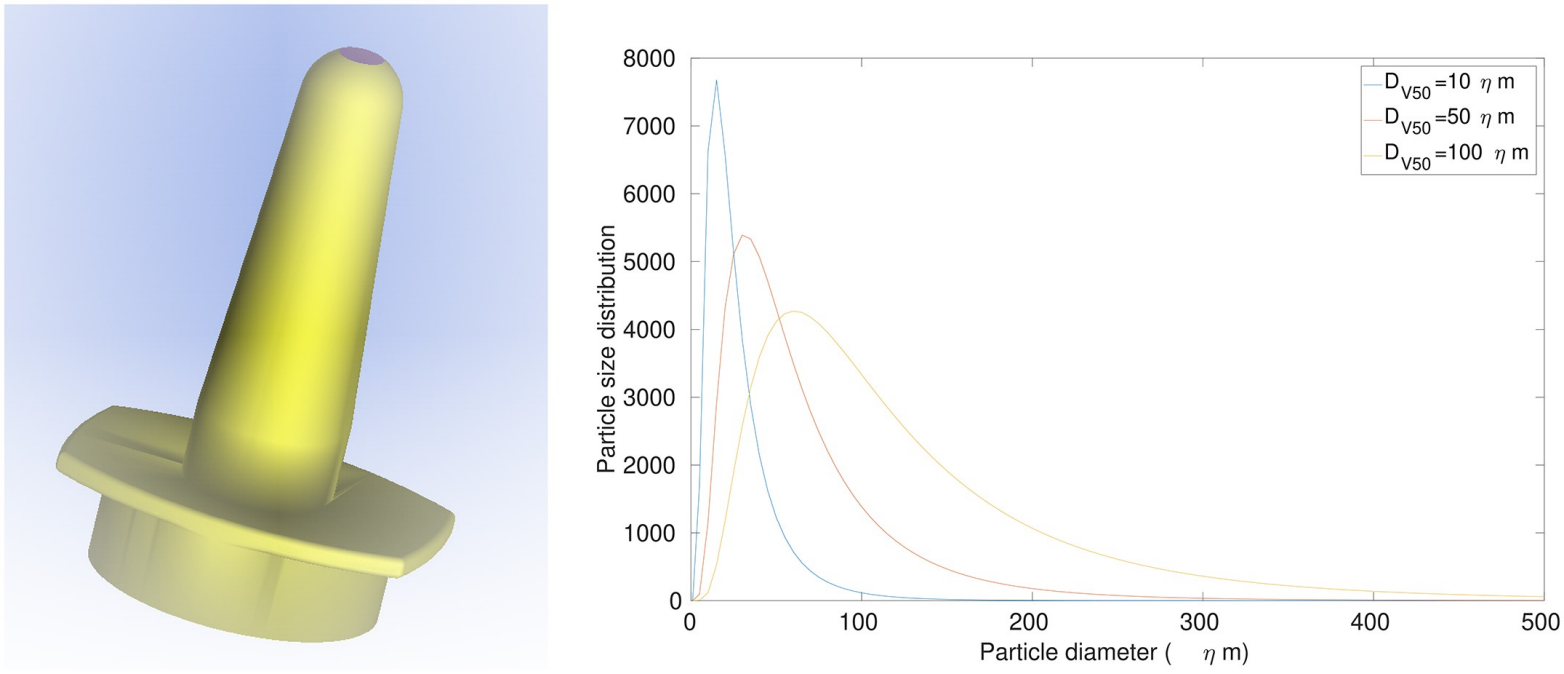
Log-normal distribution function showing the profiles for medians of *DV*50 = 10, 50, 100.

Nasal spray atomizers can be designed to change the particle size distribution. The volume median diameter of Eq 7 was changed in the range *Dv*50 = 10 − 150μm in increments of 10μm, to observe its influence on particle deposition. The resulting particle size distributions for *Dv*50 = 10, 50, 150μm are shown in Fig 4. The total number of particles released in one actuation was approximately 1 million.

**Fig 5:**
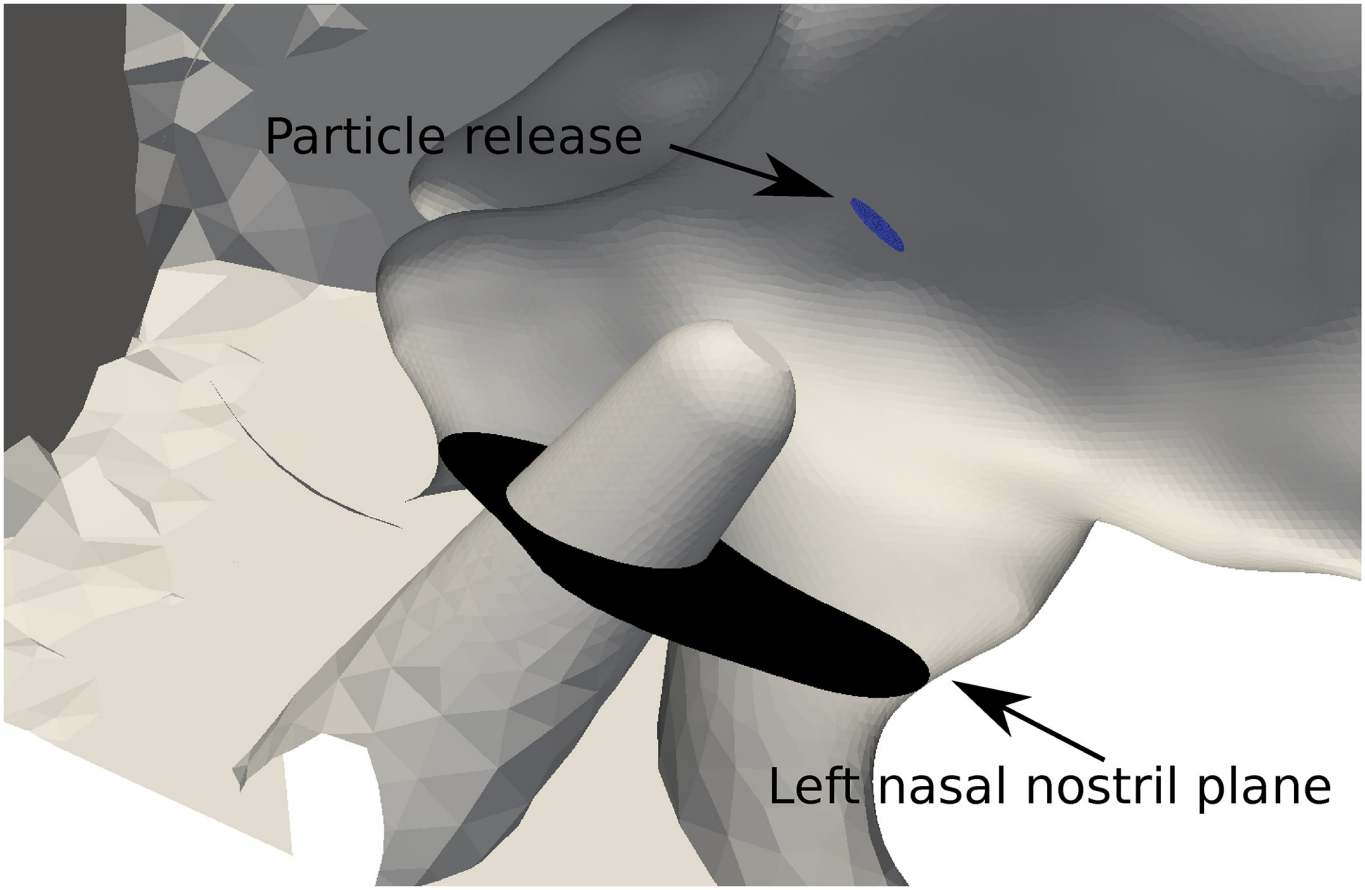
Spray insertion into nasal cavity, showing relative distances. Particles released from a break-up distance downstream from the spray nozzle.

A realistic nasal spray device based on the Flonase and Beconase devices, was included where the nozzle length was 2*cm* (Fig 5). [55] suggested that vestibular nozzle insertion in a 3D nasal model was not essential for reliable airflow simulations as the inlet perturbations (due to the nozzle placement zone) hardly affected the posterior airflow and particle transport and deposition trends. Their study used laminar flow models of flow rates based on the subject-specific allometric scaling [56], $\dot{V}=1.36M^{0.44}$ for males (sitting awake) and $\dot{V}=1.896M^{0.32}$ for females (sitting awake). Inclusion of the spray nozzle produces an occluded flow region at the nostril inlet and this leads to increased local flow acceleration. This is expected to be strengthened in the sniffing condition where the peak flowrate was 5 times of [55]. Further differences include a turbulent flow field which accounts for the stronger inlet perturbations not found in resting laminar breathing rates.

The intranasal spray guide by [57] suggests nasal sprays be directed away from the septum and towards the lateral nasal wall. The nozzle in this study was placed 0.5cm into the centroid of the left nasal nostril plane (see Fig 5) at an insertion angle of 30° to the coronal vertical axis, and 20° to the saggital vertical axis.

Particles were introduced into the computational domain from a breakup length of 3mm from the nozzle exit [51] at the start of the simulation and re-injected every 0.005 seconds until the last injection time (0.08 seconds) thus a total of 16 injections. This produced a total of 1 million particles released during the entire simulation. The number of suspended particles remaining in the domain at the end of the simulation was negligible considering the total deposited.

### Model validation

#### Analysis of grid convergence

A grid convergence study was performed for the three different mesh resolutions, i.e. a coarse mesh (M1; 2.3 million elements), a medium mesh (M2; 6.3 million elements) and a fine mesh (M3; 50 million elements) see Table 1. The fine mesh was produced using the Mesh Multiplication technique described in [58]. This technique consists in refining the mesh uniformly, recursively, on-the-fly and in parallel, inside the simulation code. For tetrahedra, hexahedra and prisms, each level multiplies the number of elements by eight, while a pyramid is divided into ten new elements. The finest mesh was obtained using a one-level mesh multiplication from the medium mesh (M2), therefore obtaining approximately (due to the presence of pyramids) eight times more elements. The time to produce this multiplied mesh in parallel is almost negligible [58].

**Fig 6:**
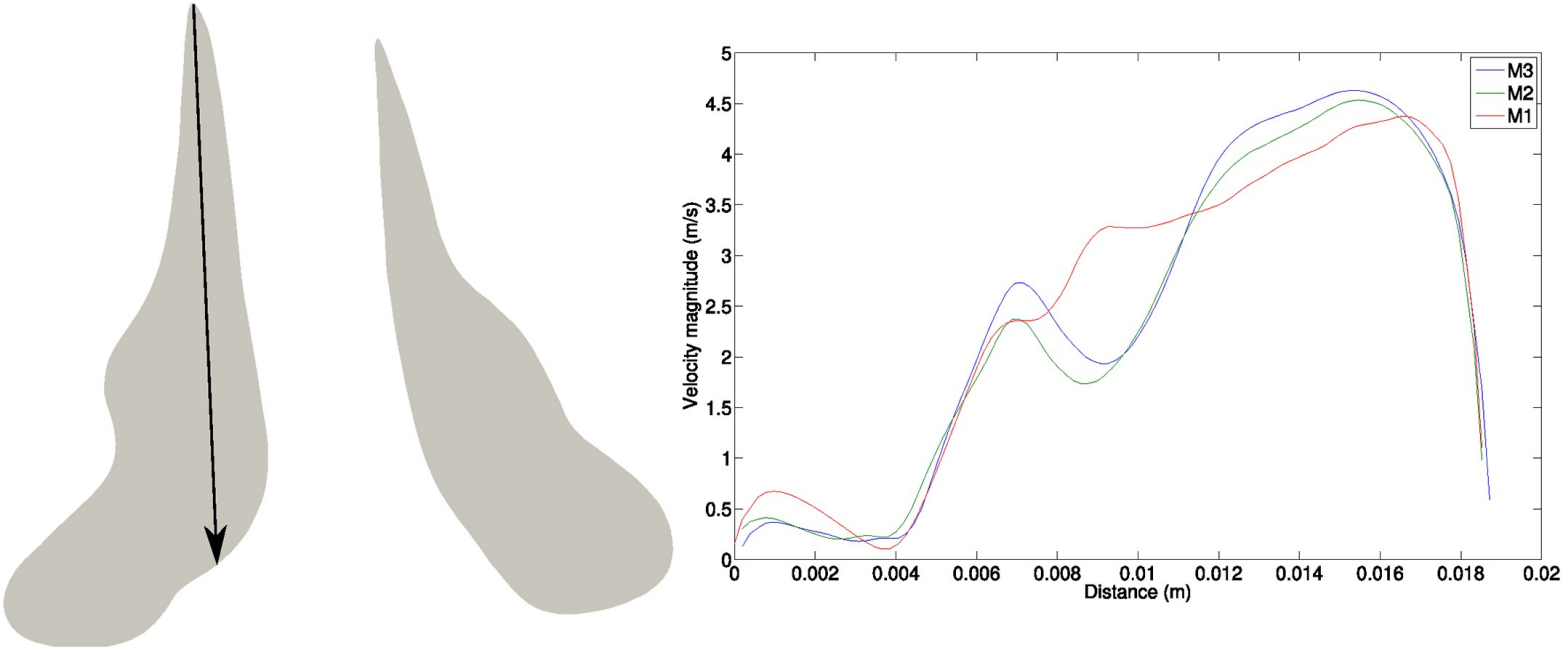
Time average velocity magnitude profile with different mesh resolutions at the sagittal plane on the slice located at 16*mm* distal from the nostril on an arbitrary line (*Q*_*max*_ = 57.4*L*/*min*).

The mean velocity magnitude profile was taken along an arbitrary line at the nasal valve slice indicated in Fig 6. The cross-section slice was 16mm from the nostril inlet, and the flow rate used was *Q*_*max*_ = 57.4L/min. The results of the velocity profiles show both meshes (M2,M3) captured the main flow trend, with only minor discrepancies between the two models. Despite the minor discrepancies, the medium meshed model (M2) with 6.3 million elements, was used for the analysis since it strikes a balance between computational costs and accuracy of solution.

**Fig 7:**
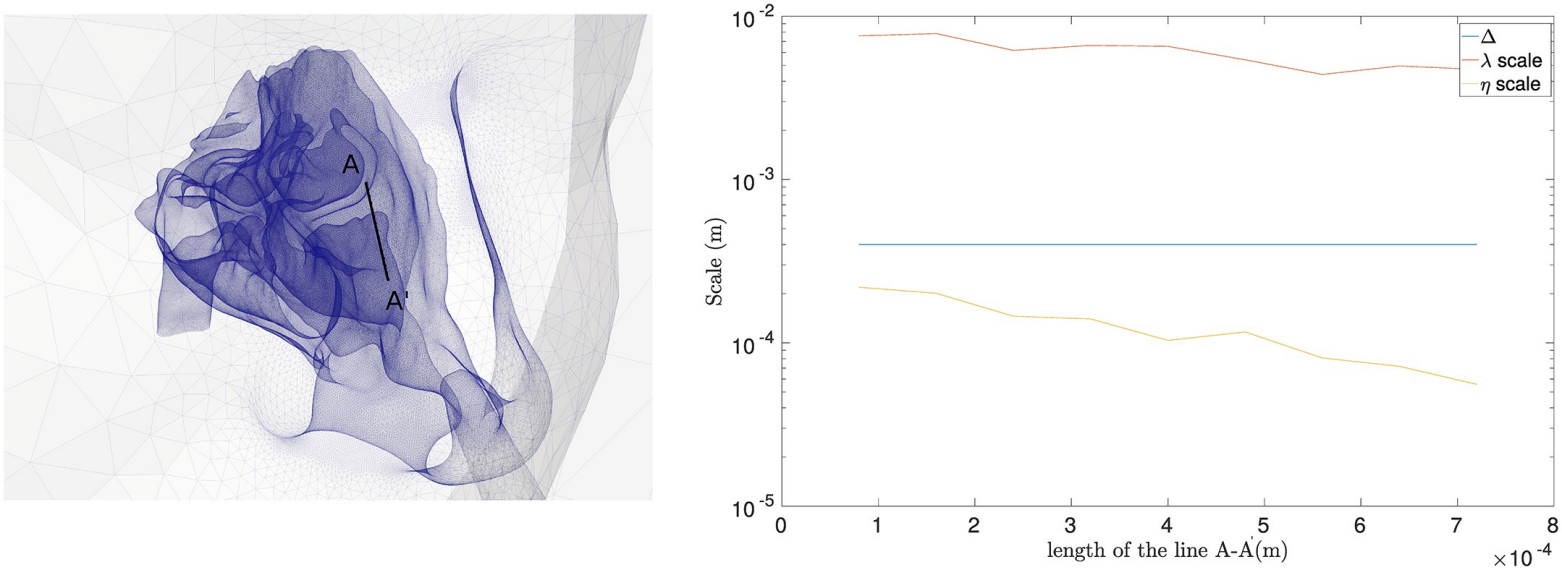
Comparison of different turbulence scales (M2 mesh and *Q*_*max*_ = 57.4*L*/*min*) on the most critical region (downstream of nasal valve).

In the LES approach, the grid resolution (Δ), which is the filter size, and time resolution (Δ*t*) are crucial to obtain a reasonable ratio between the grid size and the smallest eddies of the turbulent motions (the Kolmogorov scale, *η*). While there is no universally accepted criterion for LES grid size requirement, a ratio $\frac{\Delta }{\eta }$ less than 20 is reasonable [59]. The grid resolution should also be between the turbulent length scales of Taylor microscale (λ) and Kolmogorov scales (*η*). The Taylor microscale is used to characterise a turbulent flow and is larger than Kolmogorov scale [60]:

$$\begin{array}{c} \lambda =\sqrt{\frac{10(\nu +\nu_{sgs})k}{\varepsilon }} \end{array}$$ (8)

$$\begin{array}{c} \eta ={\left(\frac{{\left(\nu +\nu_{sgs}\right)}^{3}}{\varepsilon }\right)}^{\frac{1}{4}} \end{array}$$ (9)

where *k* is the turbulent kinetic energy, *ε* is the turbulent dissipation rate, *ν* is the fluid kinetic viscosity, and *ν*_*sgs*_ is the subgrid-scale viscosity. Fig 7 shows the grid size (Δ) of model M2 compared to the turbulence scales along line A-A‘, which is located downstream of the right nasal valve at the maximum of the sniff profile *Q*_*max*_ = 57.4L/min. The grid size was between the Taylor and Kolmogorov scale and the equivalent ratio $\frac{\Delta }{\eta }$ was 3. With this criterion we affirm that the grid is sufficiently fine for LES.

#### Particle deposition

**Fig 8:**
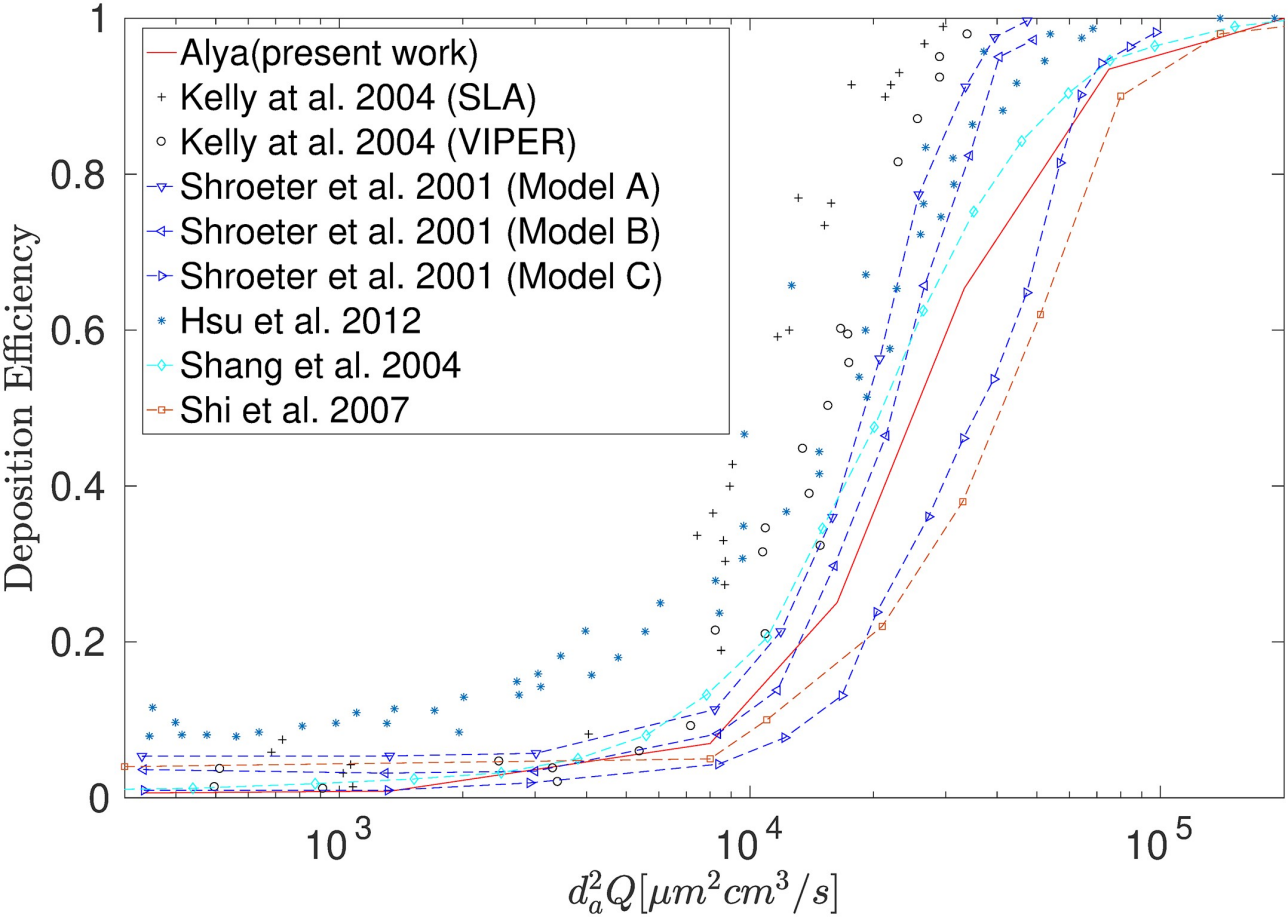
Micro particle deposition efficiency comparison between simulation and experiments.

Particle deposition in the nasal cavity was compared to experimental data reported by [61, 62] and numerical data [47, 49, 63] (see Fig 8) where the flow rate used for the comparison was a constant 20L/min. In order to standardize the results, the inertial parameter (IP) was used, i.e.

$$\begin{array}{c} IP=d_{p}^{2}\cdot Q \end{array}$$ (10)

where *d*_*a*_ is the particle aerodynamic diameter (i.e. 1g/cm^3^) and *Q* is the volumetric flow rate. Fig 8 shows good agreement between the simulation performed by the present code (Alya) and the numerical results of [47, 49, 63]. Differences in deposition results are due to the coarser airway surfaces in the replica producing higher deposition efficiencies than the numerical model, already observed frequently in literature, see [64, 65] who provide an extended study which can be summarized as the “wall roughness region enhanced particle capturing effect” or other study [66] who compared deposition of different level of surface roughness replicas (see Fig 8 Model A,B,C) from [61] with LES simulations.

#### Modelling of surface area deposition coverage

To convert deposition of a Lagrangian particle onto a surface, a formula to calculate a flux was used. A given surface *B* was considerd as composed of the union of boundary mesh elements *b* of which particles deposit onto. Setting $n_{b}^{boun}$ as the number of particles accumulated on a particular mesh element *b* and $\rho_{b}^{boun}=n_{b}^{boun}/|b|$ is the particle number density on each element, where |*b*| is the area of element *b*. To pass these boundary densities to the nodes of the mesh, a *L*^2^-projection method was used to (e.g. transfer of a discontinuous field to a continuous nodal field). Setting $\rho_{i}^{node}$ as the density of particles on each node *i*, and applying the projection produces:

$$\begin{array}{c} \sum_{b}\sum_{j}\int_{b}N_{i}N_{j}\rho_{j}^{node}db=\sum_{b}\int_{b}\rho_{b}^{boun}N_{i}db\;\forall \;i\in B, \end{array}$$

where *N*_*i*_’s are the boundary shape functions associated with the boundary nodes *i* = 1, 2, …. The left-hand side term can be lumped in order to obtain a diagonal mass matrix to solve for $\rho_{i}^{node}$ on each node *i*.

**Fig 9:**
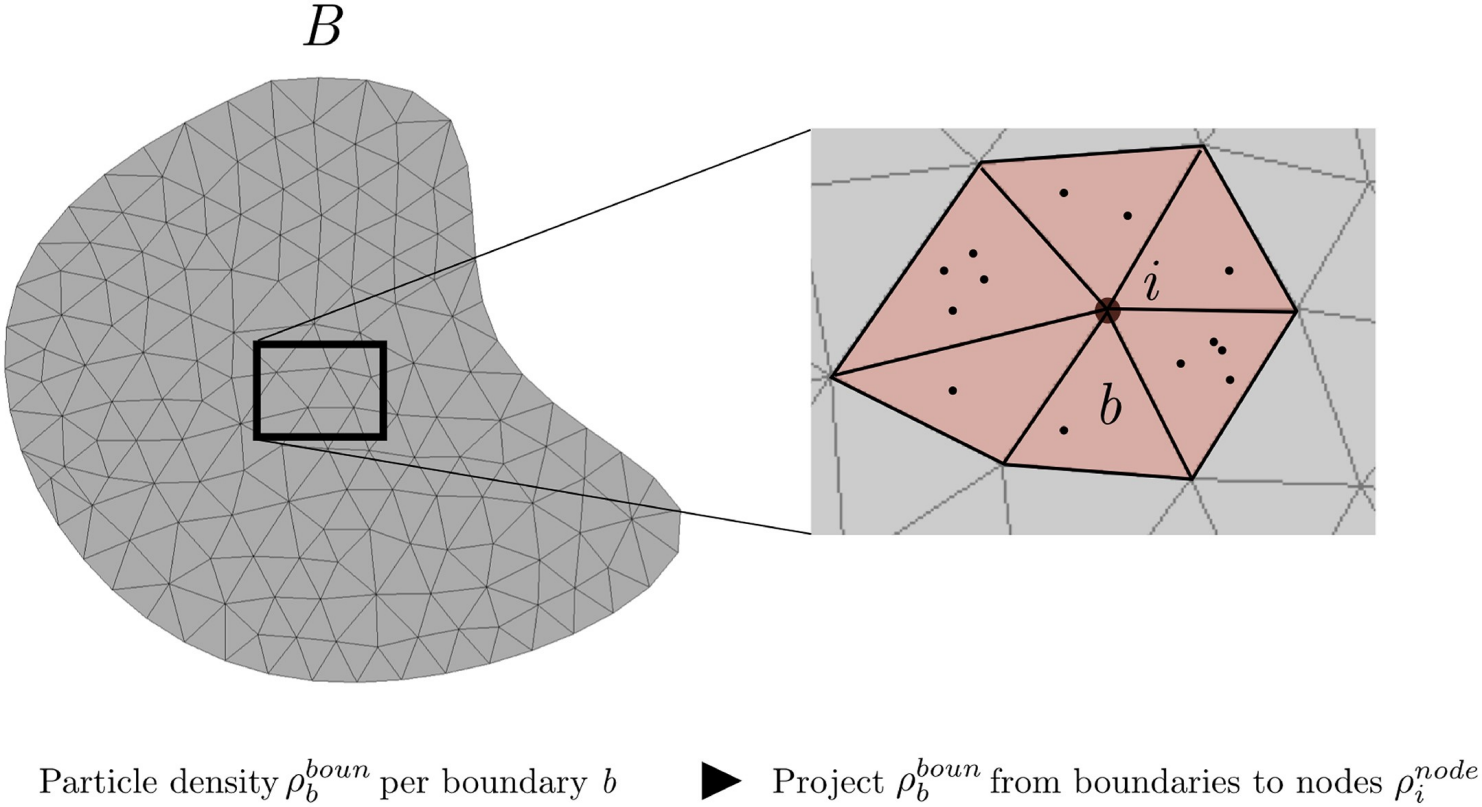
Transferring the boundary density of particles $\rho_{b}^{boun}$ to the nodes $\rho_{b}^{node}$ via a projection.

Summing over *i*, and noting that ∑_*i*_ *N*_*i*_ = 1, produces:

$$\begin{array}{c} \sum_{b}\sum_{j}\int_{b}N_{j}\rho_{j}^{node}db=n_{b}^{boun}, \end{array}$$

which conserves the total number of particles on both the boundary and nodal information (Fig 9).

#### Computational requirements

The simulations were carried out on the MareNostrum supercomputer, hosted by the Barcelona Supercomputing Center. For instance, to carry out the simulation on the MareNostrum, 110,000 time-steps are required on 480 cores requiring approximately 20h for A1, 15h for A2 and 2h for A3. In order to keep a good parallel efficiency, between 20,000 and 30,000 elements are used on each core.

## Results

### Airflow field

**Fig 10:**
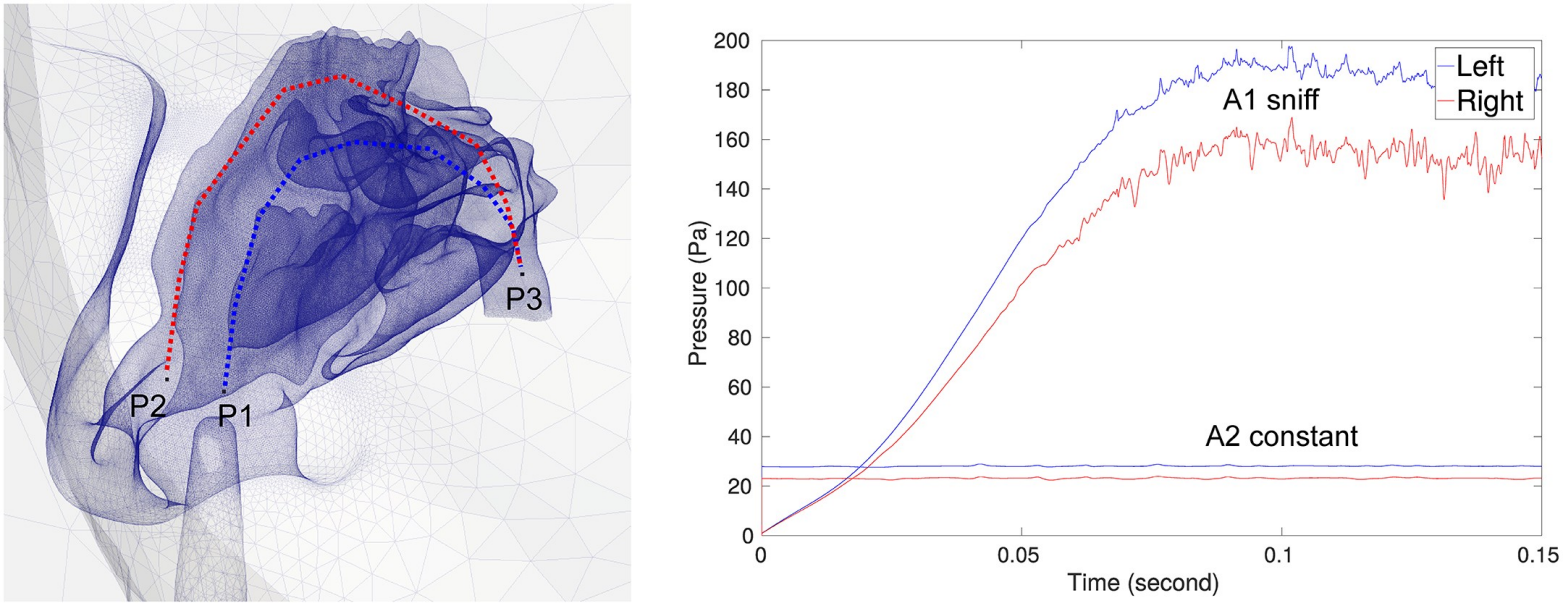
Static pressure drop across the right and left nasal cavity during time period (0.0s to 0.15s] for A1 and A2 inhalation conditions.

Fig 10 displays the pressure loss between inside the nostril to the nasopharynx in the left and right nasal cavity for both inhalation conditions. Increased resistance was found in the left nostril due to the nozzle which occluded the nostril area and this led to higher pressure loss in the left chamber for both inhalation conditions. The pressure drop between left and right chamber for sniff breathing (time period of 0.1s to 0.15s) was 30Pa, which was six times larger than for the constant flow rate that was 5Pa. The sniff peak flow rate during time 0.1s to 0.15s was approximately 2.85 times larger.

The oscillations observed in Fig 10 for the sniff inhalation condition are produced by flow features occurring downstream of the nasal valve and instabilities in the main passage way. A key feature of the transitional flow downstream of the nasal valve is the vortex shedding and flapping motion of the shear layer, located on the boundary between the nasal valve jet and the superior separated flow region, which was reported in [32, 46].

**Fig 11:**
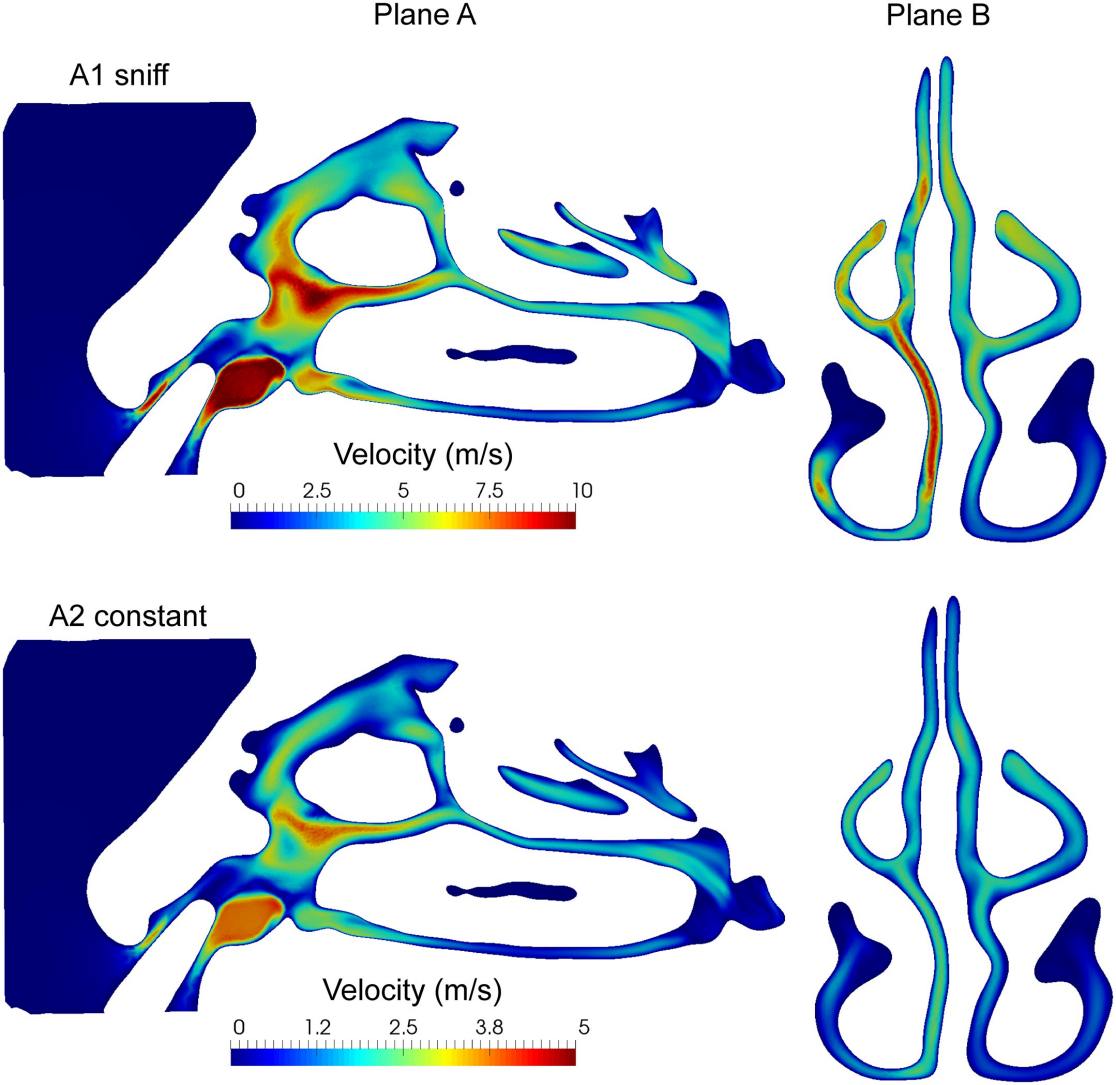
Mean velocity of sagittal plane of left nasal cavity (plane A) and coronal plane in the middle of nasal cavity (plane B) computed for the time period 0.1s to 0.15s for A1 and A2 inhalation conditions.

**Fig 12:**
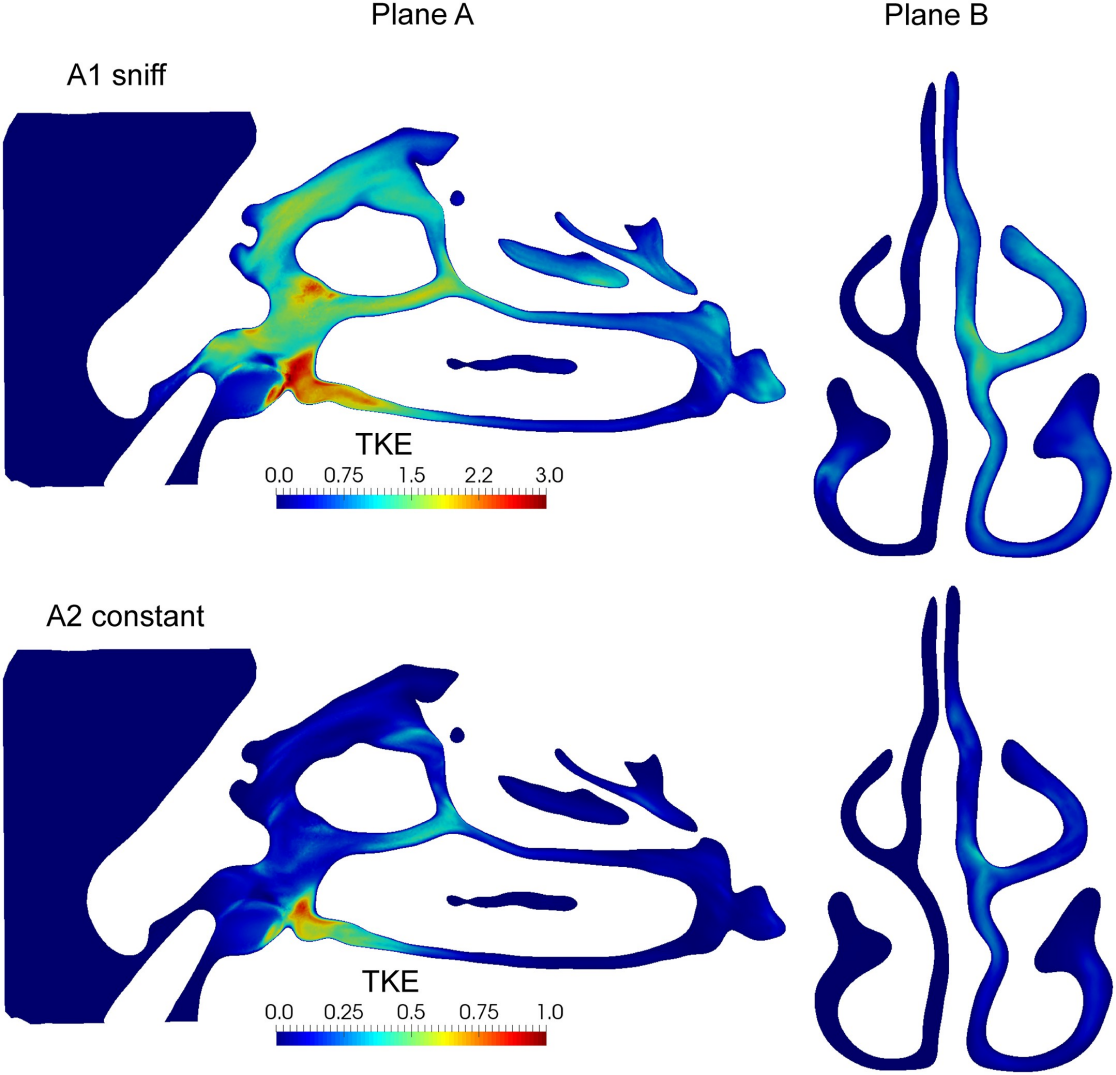
Turbulent kinetic energy (m^2^/s^2^) of sagittal plane of left nasal cavity (plane A) and coronal plane in the middle of nasal cavity (plane B) computed for the time period [0.1-0.15s] for A1 and A2 inhalation conditions.

The time-averaged velocity flow field provides an overview of persistent flow features and flow distribution; while temporal fluctuations describe the turbulent kinetic energy (TKE). The mean flow and TKE was obtained in plane-A and plane-B, (Figs 11 and 12).

The mean velocity for sniff inhalation was approximately two times higher than for the constant flow. Despite this difference, complex flow patterns caused by the nozzle insertion were found under both inhalation conditions (see Plane A of Fig 11). In particular, there is high velocity and recirculation found in the vestibule and the nasal valve regions. In the coronal section (plane B), flow occurs through the middle of the nasal cavity for both inhalation conditions and there is higher velocity through the right chamber. The uneven flow distribution is more pronounced for the sniff inhalation condition where the inter-chamber difference in pressure was 30 Pa.

Fig 12 shows velocity fluctuations on the sagittal/coronal sections where the order of magnitude is more than twice as large for sniffing condition compared with constant inhalation. The sagittal view (plane A) showed high TKE values in the vestibule and the nasal valve. The presence of the nozzle produced local unsteady flow fluctuations downstream, as the consequence of the left nostril area reduction and the complex geometry in the anterior nasal cavity. The coronal view (plane B) highlights the TKE difference between left/right chambers way and the difference in turbulent energy for both inhalation conditions. There is a non-negligible value of TKE on the left chamber particularly for the sniff (A1), which is a result of the upstream flow. The flow fluctuations occurring in the left vestibule gradually decreased in intensity as it is convected through the nasal cavity.

### Particle deposition

#### Regional deposition

**Fig 13:**
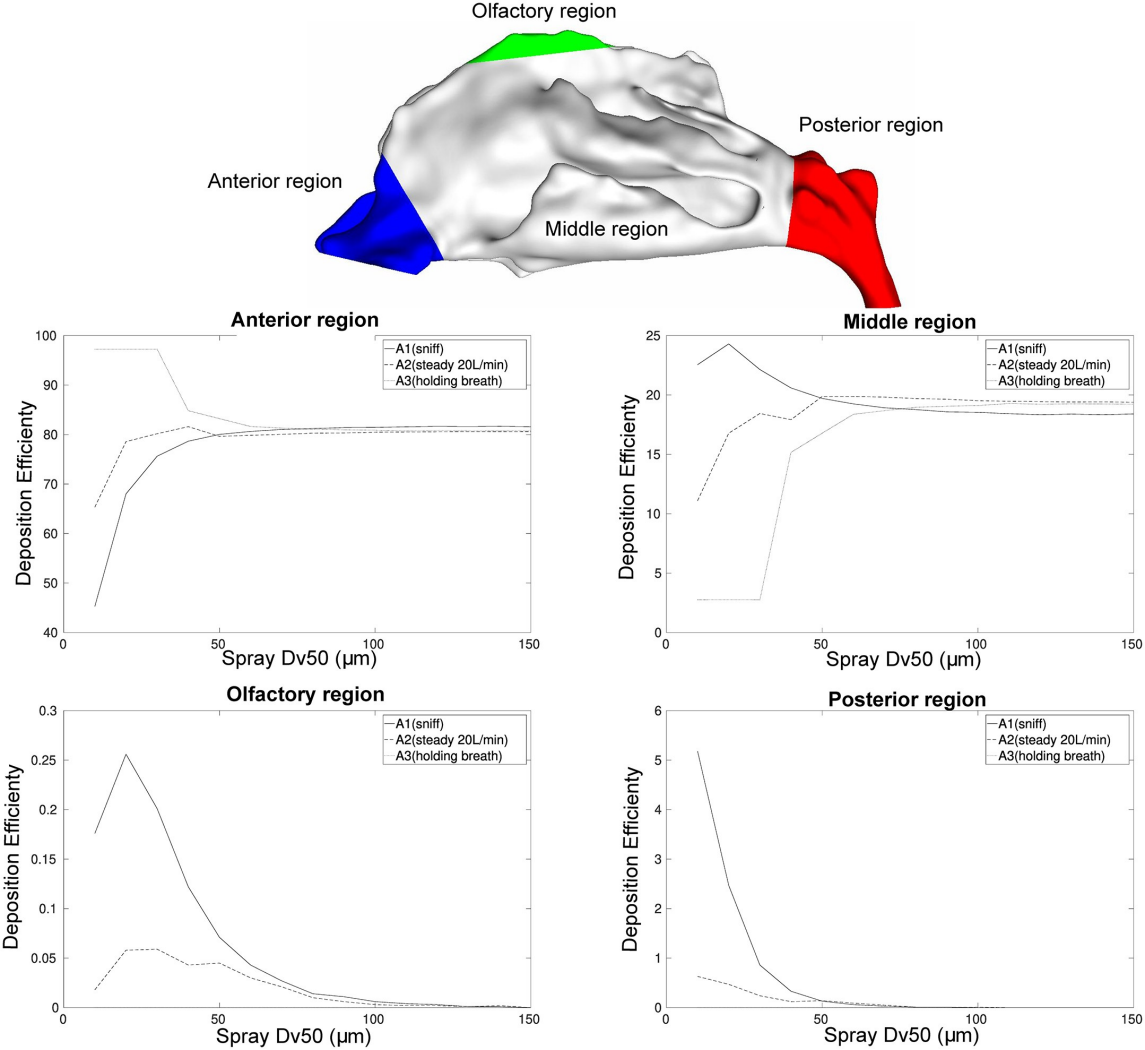
Regional deposition efficiency for the three inhalation conditions in function of particle size distribution *Dv*50.

Sprayed particle deposition across the nasal cavity regions was affected by flow profile and particle size distribution (Fig 13). When *Dv*50 > 50μm deposition in the anterior region was stable at 80%. However, when *Dv*50 < 50μm anterior deposition decreased for sniff and steady inhalation, while it increased for a breath hold (A3) reaching a peak deposition of 98%. A similar trend was found in the middle nasal cavity region, where *Dv*50 > 50μm produced relatively constant deposition efficiency of 18-22% for all breathing profiles. For *Dv*50 < 50μm a sniff condition increased deposition, while constant breath, and breath hold reduced the particle deposition. For *Dv*50 = 10μm there was increase in deposition in the posterior region which suggests particles passed through the anterior region, it also passed through the main nasal passage, and increased deposition in the posterior region. This is attributed to a large proportion of the particle size distribution behaving with non or low inertial properties, typically *D*_*p*_ < 5μm.

In the posterior region, the sniff condition transported particles deep enough for deposition, although this influence was limited to particle size distributions with *Dv*50 < 50μm. The constant breath exhibited a small number of deposited particles (< 1%) while no particles deposited in this region during breath hold. In all breathing cases, deposition in the olfactory region was very minimal with less than 0.3% deposition. This suggests the settings used to define the sprayed particle conditions are not suitable for attempting targeted drug delivery to the brain via the olfactory bulb.

#### Deposition pattern

**Fig 14:**
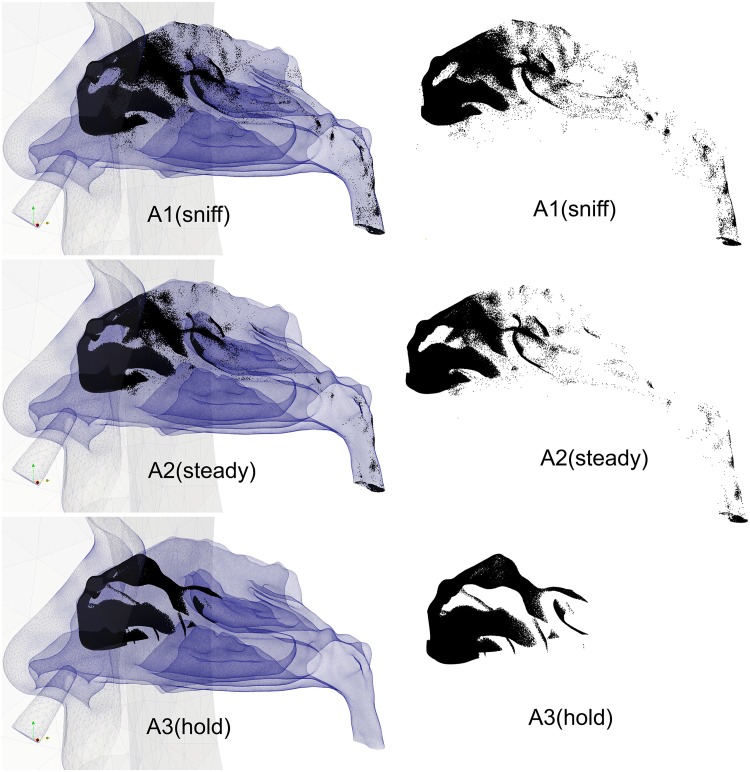
Example of deposition pattern for the three inhalation conditions.

The deposition pattern for particle size distribution of *Dv*50 = 50μm is similar for all breathing profiles − where the deposition region is inline with the direction of the nozzle. The location is superior to the main nasal passage opening, and just anterior of the olfactory region. Since micron particle deposition has strong dependence with the inertial properties of the particle, it is evident that the deposition pattern is a consequence of this. From Fig 8, deposition of *D*_*p*_ > 25μm produced > 99% deposition efficiency. The particle size distribution in Fig 14 exhibited a larger proportion of particle diameters greater than *D*_*p*_ > 25μm. The effect of convection caused by inhalation is evident from the particle transport through the nasal passage. The sniff condition produced greater scattering of particles followed by the constant inhalation, while the breath hold had no convective influence.

#### Deposition penetration

**Fig 15:**
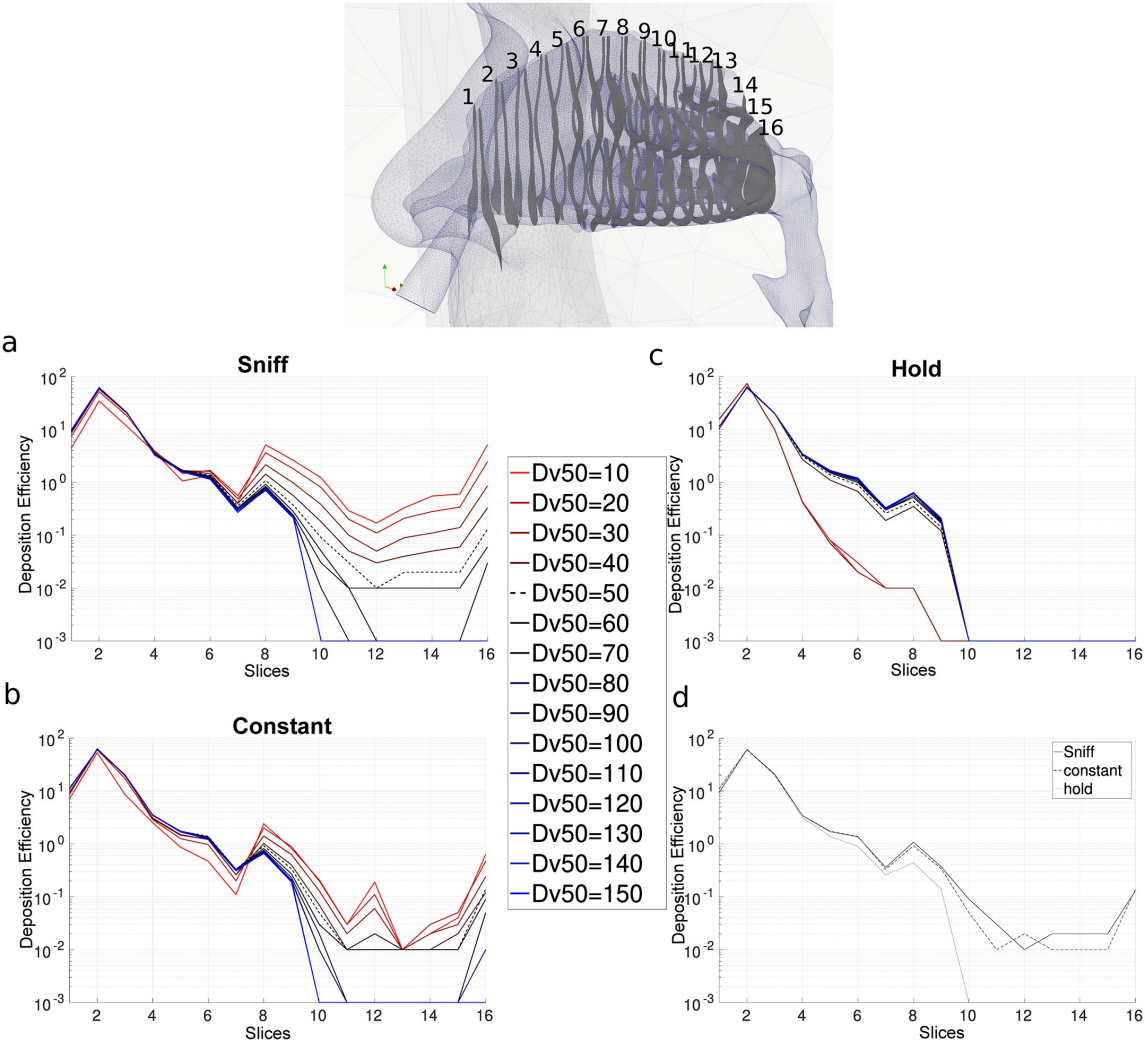
Particle deposition efficiency as a function of slice position; (a) to (c) corresponding to A1, A2 and A3 inhalation condition then (d) is the comparison of the three profile for *Dv*50 = 50μm.

There is no established definition of where the anterior, middle, posterior boundaries are and in many cases these are arbitrarily defined. Therefore these boundaries can bias the reported deposition efficiency of the anterior and middle regions when there is high deposition clustered in the anterior half of the nasal cavity. The deposition pattern in Fig 14 shows a near continuous region of particle deposition from the vestibule through to the middle nasal cavity region, and therefore deposition penetration can be used to demonstrate sprayed particle performance (Fig 15). This was defined as the particles depositing in the regions between each slice in the axial flow direction. In all breathing conditions, peak deposition occurred between Slice 1 and 2. From Slice 2 to Slice 6, the deposition gradually decreased and a local maximum was found at Slice 7 to 8. This local maximum was caused by the presence of the turbinates in the airway passage increasing the local deposition (see plane B defined in Fig 1). The deposition curves show *Dv*50 < 50μm (red curves) had less deposition than *Dv*50 > 50μm (blue curves) between slice 1-7. This was the opposite for slice 7-16 where the *Dv*50 < 50μm had greater deposition than *Dv*50 > 50μm. With sniff and constant breath flow profiles Fig 15(a) and 15(b) respectively the curves approximately collapsed until slice 7 then diverged, the logarithmic setting emphasises these differences.

#### Surface area deposition coverage

Targeted drug delivery for systemic action is most effective when drug particles can penetrate the highly vascularized main nasal passage surface. The surface area deposition coverage showed that when *Dv*50 > 60μm the surface deposition was approximately 6cm^2^, while for *Dv*50 < 60μm, the results exhibited increased deposition from sniffing, and constant flow breathing conditions with a maximum surface deposition of approximately 16cm^2^ for *Dv*50 = 20μm. This is nearly three times the surface area coverage which could improve the therapeutic efficacy of drug delivery.

#### Optimal configuration

To evaluate the best configuration of the nasal spray and inhalation condition an equation for an optimal combination was developed. Two parameters of interest are possible to define (P1 and P2) where a weighting (W1, and W2) is included for each parameter summing to a value of one, defined in Eq 11.

$$\begin{array}{c} X=W_{1}*(1-\frac{Max\left(P_{1}\right)-P_{1}\left(D_{V50}\right)}{Max(P_{1})})+W_{2}*(1-\frac{Max\left(P_{2}\right)-P_{2}\left(D_{V50}\right)}{Max(P_{2})}) \end{array}$$ (11)

**Fig 16:**
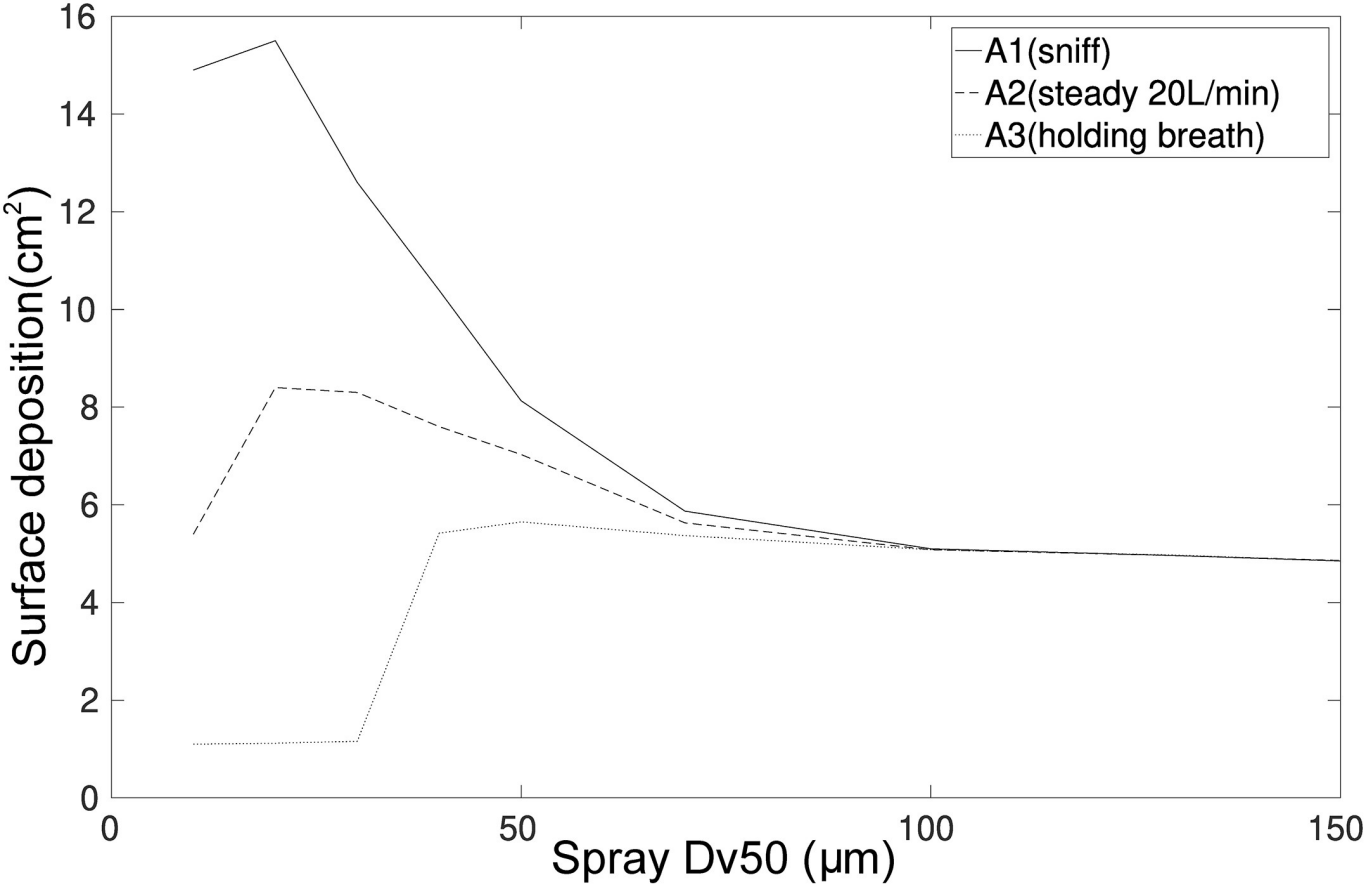
Surface deposition observed in the middle section, see Fig 16, for the three inhalation conditions.

As an example of its application, Eq 11 was calculated for all volume median diameters, *Dv*50 and inhalation conditions, A1, A2, and A3. The two parameters chosen where P1 = surface area deposition coverage and P2 = deposition efficiency in the olfactory region (P2). For single parameters, W1 (or W2) is set to 1.0, and the remaining parameter is set to 0. The single parameter was not given as since the analysis can be deduced from Figs 13 and 16. For the multi-parameter analysis, W1 = W2 = 0.5, although either weighting could be skewed up to a value of 1.0. The results are given Table 2 which shows that a *Dv*50 = 20μm under sniffing condition (A1) provided the most effective strategy for maximum coverage deposition in the main nasal passage and a maximum deposition efficiency in olfactory region.

**Table 2:** Optimal configuration mixing surface area deposition coverage and deposition efficiency in the olfactory region.

*D*_*V* 50_	10	20	30	40	50	60	70	80	90	100	110	120	130	140	150
A1	0.82	1.00	0.80	0.57	0.40	0.31	0.24	0.20	0.19	0.18	0.17	0.16	0.16	0.16	0.16
A2	0.21	0.38	0.38	0.33	0.31	0.25	0.22	0.19	0.18	0.17	0.17	0.16	0.16	0.16	0.16
A3	0.04	0.04	0.04	0.17	0.18	0.18	0.17	0.17	0.17	0.16	0.16	0.16	0.16	0.16	0.16

## Discussion

Studies of drug particle deposition have predominantly presented results for steady flow rates for mono-disperse particles. As a result the inertial parameter is used to characterise the inertial effect of mono-disperse micron particles in the nasal cavity which is a function of particle diameter, and constant inhalation flow rate. An inertial parameter of 10^5^ (see Fig 8) was identified as the cut-off/peak value for which 100% deposition efficiency occurred. This corresponds to cutoff particle diameters of 25μm to 10μm for constant flow rates of 10L/min to 50L/min, as the minimum particle diameter for 100% deposition efficiency in the nasal cavity. To expand on the current knowledge base, this study investigated three flow profiles that represented possible inhalation conditions relevant to nasal spray administration: sniff inhalation (A1), constant flow (A2), and breath hold (A3). In addition, atomized particles were presented as a polydisperse set of particles, in the form of a distribution function. [5] described atomized nasal spray droplets using log-normal distributions with peaks between 50 − 70μm.

The results in this study provides new deposition profiles for transient behaviour of log-normal polydisperse particles under different breathing patterns. Interestingly, a cutoff particle size distribution was observed. For *Dv*50 > 50μm, the deposition efficiency was relatively constant, and independent of inhalation profile. The high inertial properties of the spray produced deposition that was directly in line with the orientation of the nozzle. For *Dv*50 < 50μm there was strong dependence on the inhalation profile and this was more apparent as the distribution of particles shifted to smaller diameters, i.e. *Dv*50 = 50μm progressively decreasing to *Dv*50 = 10μm. This suggests that reporting of particle size distribution is critical when determining strategies for targeted drug delivery.

Of the three breathing conditions, the sniff inhalation exhibited the strongest convection because of it’s higher flow rate, and this reduced deposition in the anterior regions, and enhanced deposition in the main nasal passage. In the very extreme case where *Dv*50 = 10μm, a large proportion of particles were small enough to pass through the main nasal passage and a slight reduction in deposition was found in the main nasal passage, leading to posterior deposition, and possible entry into the trachea, and lung deposition. This performance appears to contradict the notion that higher flow rates contribute to greater deposition by inertial impaction. However, since the overall deposition in this study is considering a polydisperse size distribution, then the effect of an increased proportion of smaller particle diameters becomes more evident as *Dv*50 decreases from 50μm. In addition to this the particle diameter contributes to the inertial parameter by the square power, while the flow rate contributes with a linear power, thus particle size is significantly more important than flowrate.

Clinical studies by [67] showed that gentle inspiration technique improved the intranasal distribution of sprayed particles in patients with allergic rhinitis, although the study did not consider airway geometry. The test was performed on one chamber only, and compared with no breathing on the opposing chamber, thus the effect of nasal cycling or asymmetric nasal airways was not considered. CFD studies using steady laminar flow rates found an optimum inspiratory flow rate of 15.7 to 17.4 L/minute [15, 68]. This value was assumed to be the laminar flow rate at steady state, and stated that sniffing breathing would induce additional turbulence. The results of the current study showed sniffing conditions could assist in sprayed particle conditions for polydispersed particles that had *Dv*50 < 50μm. The LES turbulent approach was used in this study which has immense computational costs, and therefore is limited to perform massive parametrical studies. Further studies using lower order turbulence modelling may be able to provide further insight into the performance of nasal spray drug delivery.

Limitations of this study include the uniform initial particle velocity which in reality is subjected to velocity fluctuations. Furthermore, many nasal spray devices use a pressure-swirl atomizer [19] which implies some swirl component within the initial particle velocities. Discrete particles (non-deforming, inert, and no breakup or coalescence) were modelled with a one-way coupled assumption which provides simpler and efficient modelling. However, this may not be true in the near nozzle region of the spray device, because liquid sheets with peak mass loadings are present. Further downstream the coupling is not expected to be strong given that the concentration of particles become more disperse.

Result uncertainties are mainly physiological, physical and numerical. The physiological uncertainties come mostly from the difficulties in creating an accurate simulation scenario from patient and medical device data: the sniff flow rate, the particle distribution (size, density, shape), the geometrical setup defined from medical image, and others. Uncertainties on the physical side stem, for example; from the deposition model (deposition occurs once a particle touches the surface), the particle law of motion (selection of governing forces), the airflow-particle coupling (herein one-way), the airflow governing equations (incompressible flow with negligible heat transfer), the turbulence modeling (herein LES model), the air properties (viscosity, density), and others.

To assess the numerical uncertainties, consider the following; An exhaustive uncertainty analysis of physiological and physical aspects is almost impossible due to limited computational resources and is thus out of the scope of this paper. However, some can be treated rather easily and are commonly used in such type of simulation. On the physiological side, a particle size distribution has been considered in Section Nasal spray. Once one has assumed the physiological and physical hypothesis delineate the scope of the study, one should control numerical uncertainties as much as possible to stay within the range established by the physical setup. Regarding the airflow, this was achieved through mesh convergence, as demonstrated in Section Model validation.

## Conclusion

The deposition of polydisperse particles representing nasal spray application was performed under transient breathing profiles of sniffing, constant flow, and breath hold. Atomized particles emanating from a nasal spray device was represented by a polydisperse log-normal distribution. The spray nozzle was included in the geometry which created a realistic flow field in the anterior half of the nasal cavity. Of the three breathing profiles, the breath hold produced no transport due to the absence of and fluid convection. The sniff condition, which exhibited peak flow of 57.4L/min produced the most significant reduction on anterior deposition. For monodisperse particles, sniff conditions increases the inertial parameter of a particle. However for polydisperse particles that have large proportion of particles with low inerital properties, the increased flow and turbulence can assist the transport of particles into the main nasal passage. The inertial parameter (*d*^2^*Q*) describes monodisperse micron deposition as a function of particle size (ie. diameter) to the square power, while the flow rate is to a linear power, thus the particle size is more important.

In monodisperse particles the cutoff value for 100% deposition efficiency was an inertial parameter of 10^5^μ*m*^2^ *cm*^3^/*s*. This study showed that a cutoff value also existed for polydisperse particle deposition. Constant deposition pattern, and efficiency that was independent of flow rate and profiles, was found when the particle size distribution *Dv*50 = 50μ*m*.

The polydisperse particles under sniff conditions, produced an increase of 300% deposition in surface area coverage in the main nasal passage which is where the highly vascularised mucosal walls exist. Other targeted regions such as the olfactory region showed negligible deposition, thus the spray particle conditions is ineffective for olfactory deposition for possible drug delivery to the brain. The method presented allows any region to be targeted, and therefore an optimisation equation was given to calculate the overall performance.

This study has produced the new deposition profiles for polydisperse particles. While a large set of parameters were evaluated (e.g. 15 particle size distributions, 3 breathing profiles, 4 regional deposition locations = 180 combinations), administration of nasal spray drug delivery presents a more parameters that were not considered. The LES modelling approach is very computationally intensive which makes it challenging for a massive parametrical study. In future larger patient samples, and spray parameters can be included for such studies to be evaluated.

## Supporting information

S1 Appendix (PDF)
