# Supplementary material for: Nasal sprayed particle deposition in a human nasal cavity under different inhalation conditions
Source: PLoS One. 2019 Sep 6;14(9):e0221330. doi: 10.1371/journal.pone.0221330 (PMC6730903; doi:10.1371/journal.pone.0221330)
Supplement: S1 Appendix — (PDF) [file pone.0221330.s001.pdf]

Order	Coefficient
0	0.0015
1	0.6813
2	472.2648
3	-10790.8421
4	114269.4525
5	705483.2425
6	2736179.6777
7	-6773661.0554
8	10395525.1742
9	-9007136.2292
10	3364786.7418

Table A: Coefficient values of the 10^{th}order polynomial function

S1 Appendix

Modelling of the sniff. The 10th order polynomial function (see Eq. A) describing the temporal evolution of the flow rate is provided in Fig 3, with the coefficients of the flow defining Eq A are given in the following table (see Table A).

$$P(x) = \sum_{i=0}^{10} C_i x^i \times 1.74 \tag{A}$$
